# USP13 promotes breast cancer metastasis through FBXL14-induced Twist1 ubiquitination

**DOI:** 10.1007/s13402-023-00779-9

**Published:** 2023-02-03

**Authors:** Binggong Zhao, Wei Huo, Xiaomin Yu, Xiaoxia Shi, Linlin Lv, Yuxi Yang, Jie Kang, Shujing Li, Huijian Wu

**Affiliations:** 1 School of Bioengineering & Key Laboratory of Protein Modification and Disease, Liaoning Province, Dalian University of Technology, Dalian, China; 2 Central Hospital affiliated to Dalian University of Technology, Dalian, China

**Keywords:** Breast cancer, Tumor metastasis, De-ubiquitination, USP13, Twist1, FBXL14

## Abstract

**Purpose:**

Epithelial-to-mesenchymal transition (EMT) is an important cause of high mortality in breast cancer. Twist1 is one of the EMT transcription factors (EMT-TFs) with a noticeably short half-life, which is regulated by proteasome degradation pathways. Recent studies have found that USP13 stabilizes several specific oncogenic proteins. As yet, however, the relationship between Twist1 and USP13 has not been investigated.

**Methods:**

Co-Immunoprecipitation, GST-pulldown, Western blot, qRT-PCR and immunofluorescence assays were used to investigate the role of USP13 in de-ubiquitination of Twist1. Chromatin immunoprecipitation and Luciferase reporter assays were used to investigate the role of Twist1 in inhibiting USP13 reporter transcription. Scratch wound healing, cell migration and invasion assays, and a mouse lung metastases assay were used to investigate the roles of USP13 and Twist1 in promoting breast cancer metastasis.

**Results:**

We found that Twist1 can be de-ubiquitinated by USP13. In addition, we found that the protein levels of Twist1 dose-dependently increased with USP13 overexpression, while USP13 knockdown resulted in a decreased expression of endogenous Twist1. We also found that USP13 can directly interact with Twist1 and specifically cleave the K48-linked polyubiquitin chains of Twist1 induced by FBXL14. We found that the effect of USP13 in promoting the migration and invasion capacities of breast cancer cells can at least partly be achieved through its regulation of Twist1, while Twist1 can inhibit the transcriptional activity of USP13.

**Conclusions:**

Our data indicate that an interplay between Twist1 and USP13 can form a negative physiological feedback loop. Our findings show that USP13 may play an essential role in breast cancer metastasis by regulating Twist1 and, as such, provide a potential target for the clinical treatment of breast cancer.

**Supplementary Information:**

The online version contains supplementary material available at 10.1007/s13402-023-00779-9.

## Introduction

Breast cancer is a highly heterogeneous disease and the most common malignancy among female cancer patients worldwide, with a high incidence rate (31% of all cancers) and second only to lung cancer in mortality (accounting for 15% of all cancer deaths) in 2022 [1]. Malignant metastasis accounts for more than 90% of breast cancer-related deaths [2]. Therefore, investigating the molecular mechanisms underlying breast cancer invasion and metastasis is crucial for identifying new breast cancer therapeutic targets and reducing the lethality of malignant breast cancer. A substantial number of studies has confirmed that malignant breast cancer metastasis is often accompanied by epithelial-to-mesenchymal transition (EMT) [3, 4].

Twist1 (twist family bHLH transcription factor 1) is an efficient EMT-TF [5]. This transcription factor is a member of the BASIC HELIX-LOOP-HELIX (BHLH) protein family and plays a pivotal role in numerous physiological and pathological processes [6]. Studies on clinical samples have shown that high expression levels of Twist1 are highly correlated with cancer cell invasion and migration [7]. Furthermore, a high Twist1 expression has been found to increase cancer cell resistance to anoikis using in vitro studies [8]. Thus, high Twist1 expression can promote EMT and lead to cancer cell metastasis. Twist1 can undergo various post-translational modifications before performing its multiple physiological functions. Several residues of Twist1 can be phosphorylated by multiple protein kinases. For instance, Thr125 and Ser127, two residues in the BHLH domain, can be phosphorylated by protein kinase A (PKA) [9], whereas protein kinase B (PKB) can phosphorylate Twist1 at Ser 42 [10], T121 and S123 [11]. Additionally, three mitogen-activated protein kinases, p38, c-Jun N-terminal kinase (JNK) and extracellular signal-regulated kinase 1/2 (ERK1/2), have been reported to phosphorylate Twist1 at Ser 68 [12]. IL-6 can activate casein kinase 2 and phosphorylate Ser 18 and Ser 20 of Twist1 [13]. In addition, Tip60 can di-acetylate Twist1 at Lys 73 and Lys76 in the histone H4 mimic domain [14]. Recent studies have also shown that the WR domain is correlated to the stability of Twist1 by being subject to ubiquitination and degradation mediated by E3 ubiquitin ligases [15], such as β-TrCP1 [16], FBXL14 [17] and RNF8 [18], and to de-ubiquitination and stability mediated by de-ubiquitinating enzymes such as A20 [19], Trabid [20] and Dub3 [21]. Twist1 can also be recognized by the selective autophagy substrate p62, which triggers the degradation of Twist1 through the autophagy–lysosome degradation system [22].

USP13 (ubiquitin specific peptidase 13), a member of the de-ubiquitinating enzyme (DUB) superfamily [23], has been implicated in tumorigenesis from multiple perspectives. USP13 has been found to stabilize p53, a typical tumor suppressor by de-ubiquitinating its de-ubiquitinating protease USP10 [24]. PTEN is another tumor suppressor with a relatively longer half-life than p53. USP13 stabilizes PTEN via direct binding to and de-ubiquitinating it, followed by inhibiting PI3K/AKT signaling and tumor growth in several cancer types [25–29] and inhibiting the progression of other diseases, such as osteoarthritis [30] and idiopathic pulmonary fibrosis [31]. It has been found, however, that USP13 also stabilizes MITF, an oncogenic protein that plays a role in melanoma development by increasing MITF de-ubiquitination, subsequently resulting in reduction of MITF proteasome-mediated degradation [32]. USP13 is preferentially expressed in glioma stem cells (GSCs), and potently promotes GSC proliferation and tumor growth by inhibiting c-Myc ubiquitination and degradation, a critical transcriptional factor for maintaining GSC self-renewal and tumorigenic potential [33]. In addition, USP13 is a STING-interacting protein that catalyzes the de-ubiquitination of STING, which is critical for host defense against viruses [34]. USP13 also regulates DNA damage responses (DDR) by targeting RAP80 [35]. Although the biochemical and molecular functions of USP13 have been explored in various human cancers, including breast cancer, the oncogenic or tumor suppressor roles of USP13 in different studies has remained controversial.

Here, we investigated a potential physical/functional interaction between Twist1 and USP13 in human breast cancer. We found that USP13 promotes breast cancer metastases through Twist1 de-ubiquitination. Targeting the USP13/Twist1 axis may provide an option to treat this fatal disease effectively.

## Materials and methods

### Cell lines and culture

The use of human HEK293T, mouse 4T1/Luc and human breast cancer (T47D, ZR-75–30, SUM159PT, MDA-MB231) cells has been reported in our previous studies [36, 37]. T47D, ZR-75–30 and 4T1/Luc cells were cultured in RPMI-1640 medium (Gibco), whereas the other cells were cultured in Dulbecco's modified Eagle medium (DMEM, Gibco). All cells were cultured in a humidified atmosphere of 5% CO_2_ at 37 °C, and the media were supplemented with 10% FBS (NQBB), 100 μg/ml streptomycin, and 100 μg/ml penicillin (Solarbio).

### Antibodies and reagents

The commercial antibodies used for Western blot analysis are as follows: anti-Twist1 (1:1000, sc-81417, Santa Cruz Biotechnology), anti-USP13 (1:3000, ab109264, Abcam), anti-RNF8 (1:1000, sc-271462, Santa Cruz Biotechnology), anti-FBXL14 (1:1000, HPA053889, Sigma-Aldrich), anti-βTrcp1 (1:1000, sc-390629, Santa Cruz Biotechnology), anti-E-cadherin (1:1000, sc-8426, Santa Cruz Biotechnology), anti-N-cadherin (1:1000, sc-59987, Santa Cruz Biotechnology), anti-Vimentin (1:1000, sc-6260, Santa Cruz Biotechnology), anti-MMP9 (1:1000, sc-21733, Santa Cruz Biotechnology), anti-Ubiquitin (1:1000, sc-8017, Santa Cruz Biotechnology), anti-Flag (1:3000, GTX115043, GeneTex), anti-GFP (1:3000, GTX113617, GeneTex), anti-HA (1:3000, GTX115044, GeneTex), anti-c-Myc (1:3000, PLA0001, Sigma), anti-GST (1:3000, sc-138, Santa Cruz Biotechnology), anti-GAPDH (1:3000, GTX627408, GeneTex), anti-tubulin (1:3000, bs-0715R, Bioss), and anti-Ubiquitin K48-specific antibody (1:1000, ZRB2150, Sigma-Aldrich). Anti-Flag Affinity Gel (B23102), Poly Flag Peptide (B23111) and Protein A/G mix magnetic beads (B23202) were obtained from Bimake Chemicals. Cycloheximide (S7418) and MG132 (S2619) were obtained from Selleck Chemicals. TGF-β1 (100–21) was obtained from Peprotech.

### Plasmids and transfection

pcDNA3.1-Flag-Twist1 was kindly provided by Dr Cheng Yang (Nankai University, China), pRK-GFP-USP13 and pRK-GFP-USP13AE were kindly provided by Dr Bo Zhong (Wuhan University, China), pcDNA3.1-HA-FBXL14 was kindly provided by Dr Bin Wang (Third Military Medical University, China). The GFP-Twist1 plasmid was generated by inserting a Twist1 DNA fragment into a pEGFP-C1 vector at the BglII and BamHI sites by standard PCR using specific primers and Flag-Twist1 as template. The GST-USP13 and GST-USP13AE plasmids were generated by inserting USP13 or USP13AE DNA fragments into a pGEX-GST vector at the EcoR1and XhoI sites by standard PCR using specific primers and GFP-USP13 as template. Deletion mutants of GFP-Twist1 and GFP-USP13 were generated by standard PCR using specific primers and GFP-Twist1 and GFP-USP13 as templates separately. The USP13-promoter-Luc plasmid was constructed by inserting a 1,701 bp DNA fragment upstream of the USP13 transcriptional start site into a pGL3-basic vector at the KpnΙ and NheΙ sites. The fragment was cloned from SUM159PT cell DNA using sense primer 5′-TCTTTTCAATGGTACTGTATTTTAACC-3′ and antisense primer 5′-GGCGACGCTATTTTTAATCCAATGGCG-3′. shRNAs specific for USP13 and Twist1 were inserted into a pRNAT-U6.1/Hygro cloning vector. The sequences used were as follows: 5′-GTGATTGAGATGGAGAATA-3′ (#1), 5′-GGGAACATGTTGAAAGACA-3′ (#2) for shUSP13 and 5′-CTGAACAGTTGTTTGTGTT-3′ (#1), 5′-GGACCCATGGTAAAATGCA-3′ (#2) for shTwist1. The pcDNA3.1–3 × Flag empty vector, as well as the HA-Ub, HA-K48 and HA-K63 plasmids were used in our previous studies [36]. Cells were transfected with the corresponding plasmids using Lipofectamine 2000 (Invitrogen) following the manufacturer's specifications after the cells were grown to ~ 70%–80% confluence.

### Co-Immunoprecipitation, Western blot and chromatin immunoprecipitation assays

Co-Immunoprecipitation (CoIP), Western blot (WB) and chromatin immunoprecipitation (ChIP) assays were performed as previously described [37]. For the ChIP assay, the following primers were used: 5′-GCCACCTCCTCCTTTTCCAACAG-3′ (sense) and 5′-GCAGAGCATGTCTCCCAGCATAC-3′ (antisense) for the USP13 promoter.

### GST-pulldown, immunofluorescence and luciferase reporter assays

GST-pulldown assay and immunofluorescence (IF) were carried out as reported before [38, 39]. For luciferase reporter assays, HEK293T cells seeded in 24-well plates were transfected with the indicated plasmids overnight at a cell density of ~ 70%–80%. Luciferase reporter activity was monitored using Centro LB 960. β-galactosidase was used to normalize the relative activity. The luciferase activity was deduced from four parallel experiments.

### Total RNA extraction and qRT-PCR

Total RNAs were extracted by RNAios Plus (9109, Takara) after which reverse transcription was carried out to synthesize cDNA as previously reported [37]. Quantitative real-time PCR (qRT-PCR) assays were carried out using a LightCycler system (Roche) with a SYBR qPCR Master Mix (Q711, Vazyme). The primers used for qRT-PCR were: 5′- CGGGAGTCCGCAGTCTTA-3′ (sense) and 5′- GCTTGAGGGTCTGAATCTTG -3′ (antisense) for Twist1, 5′- GGCTCCTTCGTCCTTCTCCTCTAC-3’ (sense) and 5′- CCTGGCACTGGTACTTCTTGACATC-3′ (antisense) for Snail (snail family transcriptional repressor 1), 5′-CCTCCTCCATCTGACACCTCCTC-3′ (sense) and 5′- AGGTAATGTGTGGGTCCGAATATGC-3′ (antisense) for Slug (snail family transcriptional repressor 2), 5′- TCTTCACCGTCTCTTTCAGCATCAC-3′ (sense) and 5′-GAGGAGAACTGGTTGCCTGTAATGG-3′ (antisense) for Zeb1 (zinc finger E-box binding homeobox 1), 5′-GGTTGAATCTGACTGACGGCTCTG-3′ (sense) and 5′-TGCCCGACTCCTGGATCACTTC-3′ (antisense) for USP13, 5′- GGGTTGAACCATGAGAAGT-3′ (sense) and 5′- GACTGTGGTCATGAGTCCT-3′ (antisense) for GAPDH (glyceraldehyde-3-phosphate dehydrogenase). Relative mRNA levels were calculated using the 2^−ΔΔCT^ method [40] with GAPDH as an internal control.

### In vitro de-ubiquitylation assay

HEK293T cells were transfected with Flag-Twist1 and HA-Ub plasmids after which immunoprecipitation was carried out to obtain ubiquitinated Twist1 from the indicated cells using Anti-Flag Affinity Gel. Next, the immunoprecipitates were eluted by Poly Flag Peptide (0.5 mg/ml) after which the eluates were incubated with GST-USP13 or GST-USP13AE at 37 °C for 2 h followed by incubation at 16 °C in the presence of ATP (1 μM) overnight. Finally, the co-incubated samples were analyzed by Western blotting.

### Scratch wound healing, cell migration and invasion assays

For the scratch wound healing assay, cells were seeded (1.0 × 10^6^ cells per well) in 6-well plates. After 24 h, a pipette tip (200 μl) was used to create a scratch wound. Next, the cell cultures were washed using phosphate-buffered saline (PBS) and incubated afterwards. The wounds were photographed under a microscope at 0 and 24 h in situ to compare the number of cells that migrated into the wound areas.

Migration and invasion assays were carried out using Transwell inserts containing 8-μm polycarbonate membranes (3422, Corning). 1 × 10^4^ cells were resuspended in serum-free medium and seeded into the top chambers of Matrigel-coated (354,234, Corning) or empty Transwell inserts. The Matrigel was also diluted in serum-free medium to 5 mg/ml. The bottom compartments of the chambers contained complete medium as chemoattractant. The inserts were removed and rinsed with PBS after 24 h after which the cells on the bottom side were stained with crystal violet and evaluated by microscopy.

### Mouse lung metastasis assay

7-week-old female BALB/c mice were purchased from Liaoning Changsheng Biotechnology company and divided into four groups randomly (5 mice per group) before injection, and fed in the same environment. 2 × 10^6^ stably transfected 4T1/Luc cells in 0.2 ml normal saline were subcutaneously injected into the mice through the tail vein. After 14 days, metastatic lung foci were detected using bioluminescence imaging before all mice were sacrificed to collect the lungs. According to standard protocols, the lungs of each group were processed for Western blot and immunohistochemistry (IHC) assays and for hematoxylin–eosin (HE) staining, respectively.

#### Statistical analysis

All data were analyzed by GraphPad Prism 8.0.1 software and are presented as mean ± standard deviation (SD). Each experiment was conducted at least three times with consistent results. Statistical analyses were performed using a two-tailed t-test to evaluate statistical significance, expressed as *p*-value. Differences were considered significant when *p* < 0.05 (*p < 0.05), and extremely significant when *p* < 0.01 (**p < 0.01).

#### Data availability

The authors declare that the data supporting the findings of this study are available within the paper. The USP13 expression level analyses between normal breast tissues and different subclasses of carcinomatous breast tissues are available in ULACAN (http://ualcan.path.uab.edu/) [41], and the Kaplan–Meier curves that support the findings of this study are available in the UCSC Xena database (https://xenabrowser.net/) and analyzed by the R package survival [42] and survminer [43] tools.

#### Compliance with ethical standards

All animal experiments were performed according to the regulations set by the Ethics Committee for Biology and Medical Science of Dalian University of Technology.

## Results

### USP13 stabilizes the Twist1 protein

**Fig. 1:**
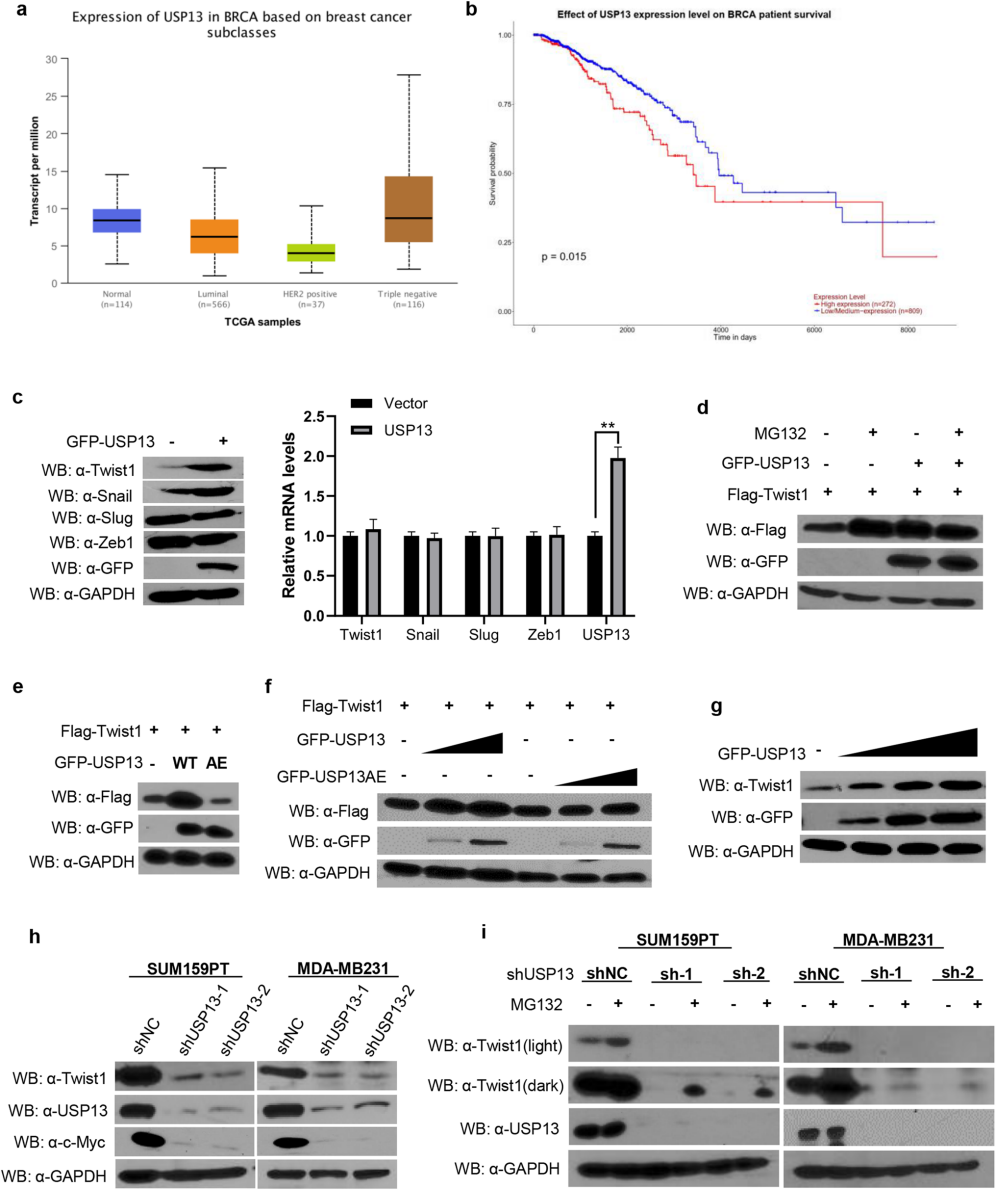
USP13 stabilizes Twist1 protein. (**a**) Expression levels of USP13 in normal breast tissues and three subclasses of breast carcinomatous tissues in the UALCAN database. (**b**) Kaplan–Meier curves obtained from the UALCAN database. (**c**) GFP-USP13 or empty vector was transfected in MDA-MB231 cells after which relative mRNA and protein levels of EMT-TFs were determined using qRT-PCR and Western blotting. (**d**) Flag-Twist1 was co-expressed with GFP-USP13 or empty vector in HEK293T cells after which the cells were treated with 20 μM MG132. Expression of Twist1 and USP13 was examined by Western blotting. (**e**) Flag-Twist1 was co-expressed with GFP-USP13 wild-type (WT) or USP13AE mutant in HEK293T cells after which the expression of Twist1 and USP13 was examined by Western blotting. (**f**) Flag-Twist1 was co-expressed with GFP-USP13 or GFP-USP13AE with increasing amounts in HEK293T cells after which the expression of Twist1 and USP13 was examined by Western blotting. (**g**) GFP-USP13 was expressed with increasing amounts in MDA-MB231 cells after which the expression of endogenous Twist1 and USP13 was examined by Western blotting. (**h**) SUM159PT and MDA-MB231 cells were stably transfected with shNC control or USP13 shRNAs after which the expression of Twist1, USP13 and c-Myc, the positive control, was examined by Western blotting. (**i**) SUM159PT and MDA-MB231 cells stably transfected with shNC control or USP13 shRNAs were treated with or without 20 μM MG132 after which the expression of Twist1 and USP13 was examined by Western blotting

In order to clarify the mechanisms underlying the mode of action of USP13 in breast cancer development, we first analyzed its expression in different breast cancer subclasses. Based on data from the ULACAN database (http://ualcan.path.uab.edu/), we found a significantly higher USP13 expression (*p* < 0.01) in highly metastatic triple-negative breast cancers compared to the other two breast cancer subclasses (Fig. 1a and Table S1). Similar results were obtained with Twist1 and it was found that it may also play a role in breast cancer metastasis. Compared with patients with low USP13 expression levels, patients with high USP13 expression levels exhibited a significantly poorer overall survival (Fig. 1b). Triple-negative breast cancer patients with high USP13 levels had the poorest overall survival compared with the other subclasses of patients (Fig. S1). In addition, we found that USP13 affected the protein levels of Twist1 and Snail in MDA-MB231 cells, but not those of other EMT-TFs (Fig. 1c).

We found that USP13, when co-expressed with Twist1 in HEK293T cells, remarkably increased the protein, but not the mRNA level of Twist1, an effect comparable to that observed after treatment with 20 μM proteasome inhibitor MG132 (Fig. 1d and Fig. S2a). This indicates that USP13 likely stabilizes Twist1 through the proteasome pathway. An enzymatic inactive mutant of USP13 (C345A/M664/739E) (designated as USP13AE) showed no effect on the Twist1 protein level, indicating that USP13 enzymatic activity is essential for Twist1 stabilization (Fig. 1e). Moreover, we found that the level of Twist1 increased in a USP13 dose-dependent manner, but not that of USP13AE, when Twist1 was co-expressed with increasing amounts of USP13 or USP13AE in HEK293T cells (Fig. 1f). The protein, but not mRNA, levels of endogenous Twist1 also gradually increased after a dose-dependent transfection of USP13 in MDA-MB231 cells (Fig. 1g and Fig. S2b), indicating that endogenous Twist1 is subject to a similar regulation. After constructing two USP13-specific shRNAs to knock down endogenous USP13 expression in SUM159PT and MDA-MB231 cells, we found that the protein, but not the mRNA, levels of Twist1 dramatically decreased in these cells, similar to those of c-Myc (Fig. 1h and Fig. S2c). In addition, we found that 20 μM MG132 treatment restored the downregulation of Twist1 in USP13-knockdown SUM159PT and MDA-MB231 cells (Fig. 1i). Based on these results, we conclude that USP13 is involved in mediating the protein stability of Twist1.

### USP13 directly interacts with Twist1

**Fig. 2:**
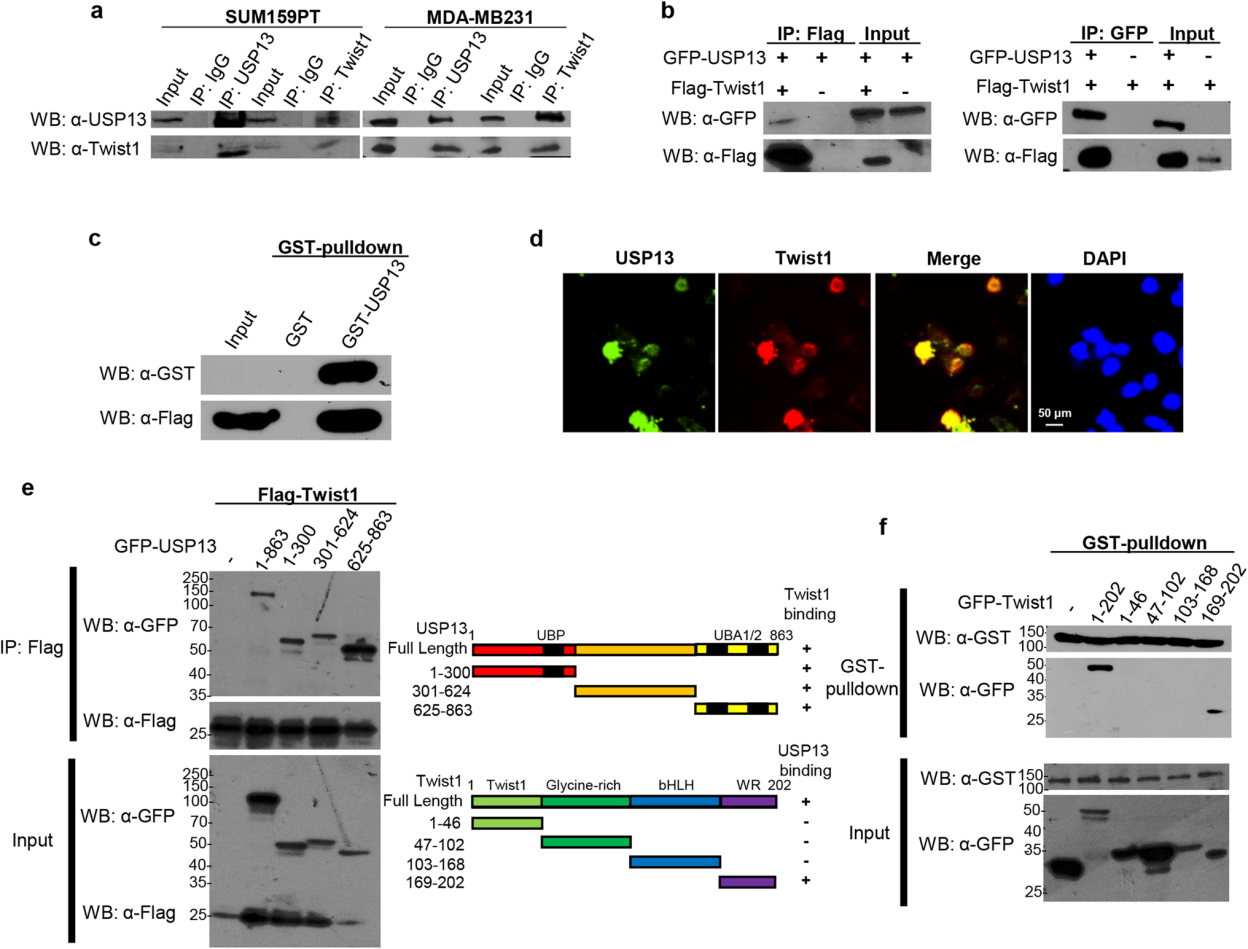
USP13 interacts with Twist1. (**a**) Endogenous USP13 and Twist1 were immunoprecipitated from MDA-MB231 and SUM159PT cells, after which bound endogenous Twist1 and USP13 were examined by Western blotting. (**b**) Flag-Twist1 was co-expressed with GFP-USP13 in HEK293T cells. Cell lysates were immunoprecipitated with either anti-Flag or anti-GFP antibody, after which the associated proteins were examined by Western blotting. (**c**) GST pulldown assays. (**d**) After fixation and staining, the subcellular localization of Twist1 (red) and USP13 (green) was visualized using fluorescence microscopy in MDA-MB231 cells. (**e**) Schematic diagram of full-length USP13 and deletion constructs. Interactions between Twist1 and USP13 domains are indicated by plus signs ( +), and detected via Western blotting. (**f**) Schematic diagram of full-length Twist1 and deletion constructs. The interactions between USP13 and Twist1 domains are indicated by plus signs ( +), and detected via GST-pulldown

In accordance with the observed association between USP13 and Twist1, we detected an efficient co-precipitation between endogenous USP13 and Twist1 in SUM159PT and MDA-MB231 cells (Fig. 2a). It also turned out that GFP-USP13 and Flag-Twist1 could interact with each other in HEK293T cells (Fig. 2b). Using GST-pulldown assays, we found that Flag-Twist1 co-precipitated with GST-USP13, but not with GST (Fig. 2c). The interaction between USP13 and Twist1 was further confirmed by IF analysis since we found that USP13 co-localizes with Twist1 in the nuclei of MDA-MB231 cells (Fig. 2d). Additionally, we found that all three GFP-USP13 domains interacted with Twist1. One of the domains, GFP-USP13 (625–863), interacted most saliently, while the other two domains increased the expression level of Twist1 versus control (Fig. 2e). Through interaction region mapping, we conclude that the N-terminal part of USP13 may contain a primary interaction domain for Twist1, and that the C-terminal part of USP13 may contain a primary de-ubiquitination domain for Twist1. Moreover, we found that GST-USP13 mainly interacted with the full length (FL) and the 169–202 amino acid fragment (Twist1 WR domain) of GFP-Twist1 rather than with other Twist1 fragments (Fig. 2f). Therefore, the above findings indicate a direct interaction between USP13 and Twist1.

### USP13 deubiquitinates Twist1

**Fig. 3:**
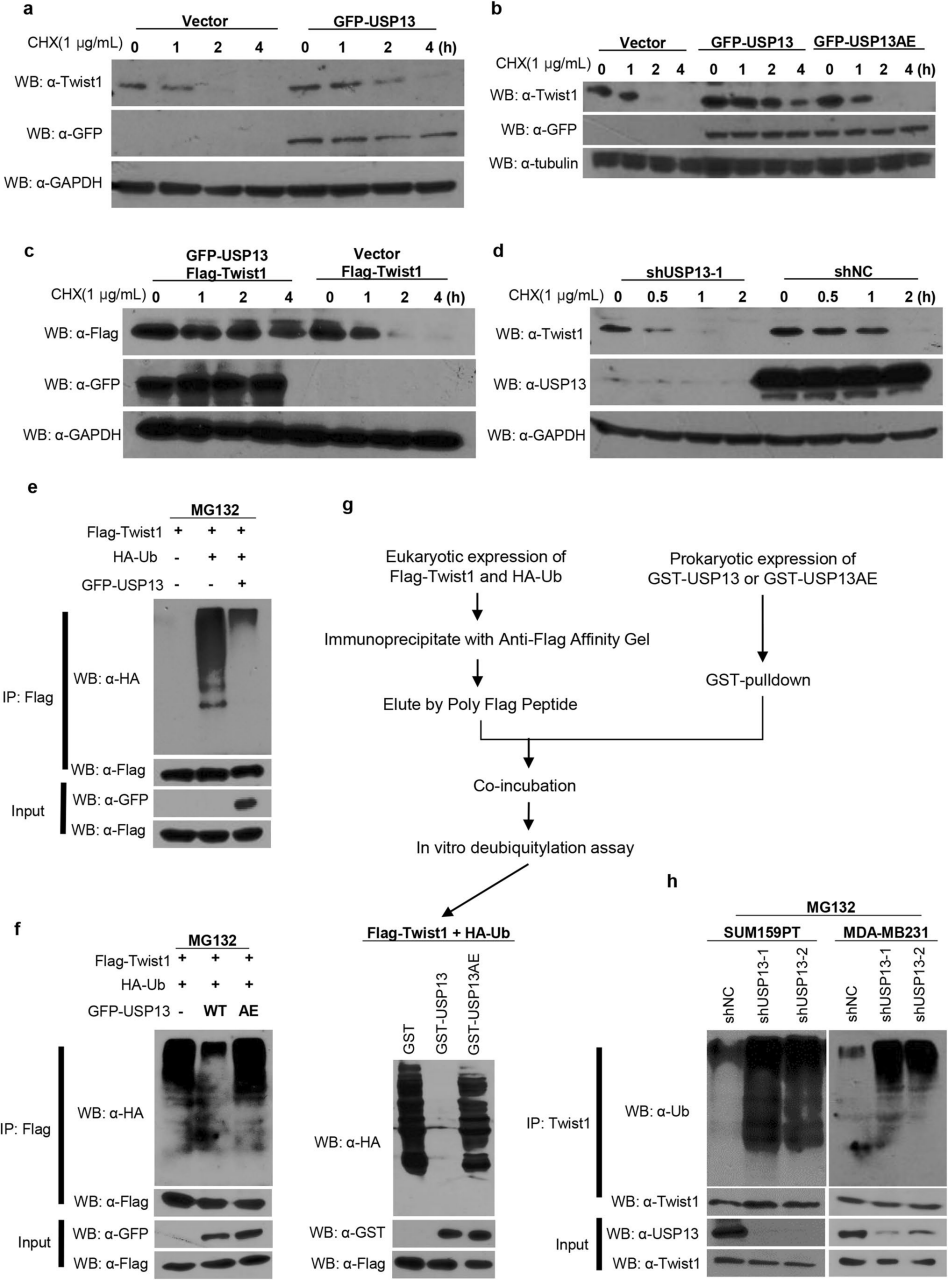
USP13 de-ubiquitinates Twist1. (**a, b**) SUM159PT cells stably transfected with GFP-USP13 or GFP-USP13AE were treated with cycloheximide (CHX) for the indicated time intervals. Expression of endogenous Twist1 and USP13 was examined by Western blotting. (**c**) Flag-Twist1 was co-expressed with GFP-USP13 or empty vector control in HEK293T cells. After CHX treatment, expression of Twist1 and USP13 was examined by Western blotting. (**d**) SUM159PT cells stably transfected with USP13 shRNA or shNC control were treated with cycloheximide (CHX) for the indicated time intervals. Expression of endogenous Twist1 and USP13 was examined by Western blotting. (**e, f**) Flag-Twist1 was co-expressed with HA-ubiquitin and either GFP-USP13 or GFP-USP13AE in HEK293T cells. Next, the cells were treated with 20 μM MG132, cell extracts were immunoprecipitated with anti-Flag antibody and poly-ubiquitination of Twist1 was examined by Western blotting. (**g**) Western blot of in vitro de-ubiquitylation assays. (**h**) SUM159PT and MDA-MB231 cells stably transfected with control or USP13 shRNAs were treated with 20 μM MG132 after which cell extracts were immunoprecipitated with anti-Twist1 antibody and poly-ubiquitination of Twist1 was examined by Western blotting

To determine the effect of USP13 on the protein stability of Twist1, we transfected SUM159PT cells with USP13 or an empty vector control and treated the cells with cycloheximide (CHX) at different time intervals to block new protein synthesis. Compared with the empty vector control group, we found that the protein level of Twist1 was significantly stabilized in the presence of USP13 (Fig. 3a and Fig. S2d), rather than USP13AE (Fig. 3b and Fig. S2e). Exogenous Twist1 was also stabilized when co-expressed with USP13 in HEK293T cells (Fig. 3c and Fig. S2f). Likewise, based on CHX chase experiments, we found that Twist1 became unstable and degraded rapidly upon knockdown of USP13 in SUM159PT cells (Fig. 3d and Fig. S2g). In Flag-Twist1 expressing HEK293T cells treated with 20 μM MG132, co-IP of Flag revealed a heavy ubiquitination of Twist1, while co-expression of USP13 almost completely eliminated it (Fig. 3e). However, co-expression of USP13AE failed to abolish ubiquitination of Twist1 (Fig. 3f). As expected, in vitro deubiquitylation assays directly showed that USP13, but not USP13AE, acted as a de-ubiquitinase for Twist1 (Fig. 3g). In contrast, we found that the level of endogenous Twist1 ubiquitination showed a sharp rise upon USP13-knockdown in SUM159PT and MDA-MB231 cells (Fig. 3h). These findings collectively indicate that Twist1 is subject to USP13-mediated de-ubiquitination, which is conducive to Twist1 protein stabilization.

### USP13 inhibits FBXL14-induced Twist1 ubiquitination

**Fig. 4:**
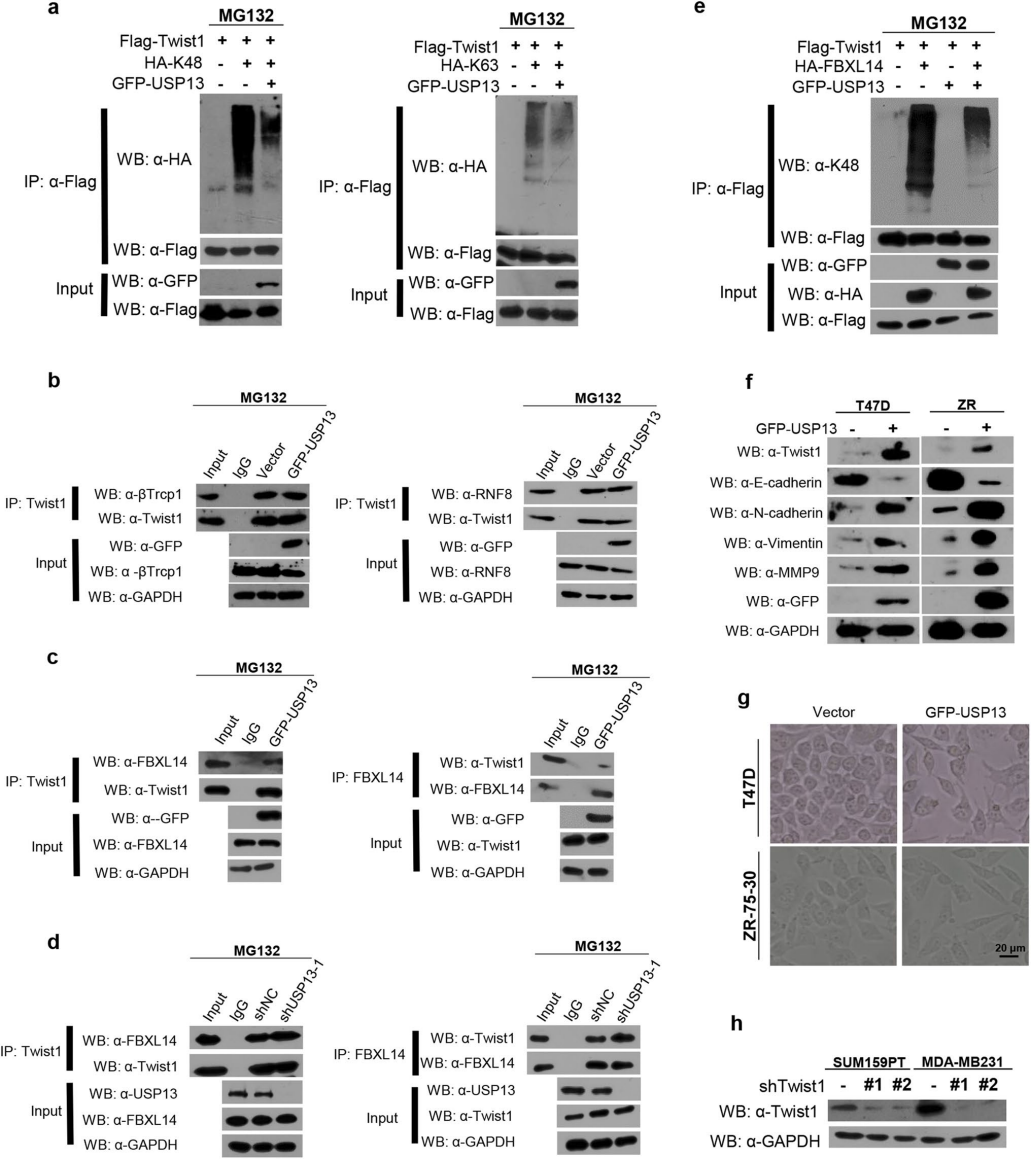
USP13 inhibits FBXL14-mediated Twist1 ubiquitination. (**a**) Flag-Twist1 was co-expressed with HA-K48 or HA-K63 in HEK293T cells after which cell lysates were immunoprecipitated with anti-Flag antibody and ubiquitination bands were examined by Western blotting. (**b**) SUM159PT cells were transfected with GFP-USP13 and treated with 20 μM MG132 after which cell lysates were immunoprecipitated with anti-Twist1 antibody and Western blotting was performed. (**c**) SUM159PT cells were treated as indicated, after which cell lysates were immunoprecipitated using anti-FBXL14 or anti-Twist1 antibodies, and then Western blotting was performed. (**d**) SUM159PT cells were transfected with shNC control or USP13 shRNA and treated with 20 μM MG132 after which cell lysates were immunoprecipitated and analyzed. (**e**) HEK293T cells were co-transfected with the indicated plasmids and, after Flag-Twist1 immunoprecipitation, K48 ubiquitination was detected. (**f**) GFP-USP13 was transfected in T47D and ZR-75–30 cells after which the indicated proteins were examined by Western blotting. (**g**) Morphology of T47D and ZR-75–30 cells transfected with empty vector or GFP-USP13. Scale bar = 20 μm. (**h**) Western blot of Twist1-specific shRNAs in the indicated cells

In light of the known capability of Twist1 to link K48 and K63 ubiquitin chains, we speculated that USP13 may be able to deubiquitinate K48 or K63 ubiquitinated Twist1. In fact, we found that USP13 overexpression caused a drop in K48 levels, but not of K63 ubiquitination on Flag-Twist1 (Fig. 4a and Fig. S2h-S2i). As mentioned earlier, recent studies have shown that several proteins may induce ubiquitination of Twist1 [16–18]. In order to explore whether USP13 determines the binding ratio of Twist1 to those proteins, we found that USP13 overexpression had no effect on the binding of Twist1 to β-TrCP1 or RNF8 in SUM159PT cells (Fig. 4b). Interestingly, however, we found that USP13 was critical for the binding of Twist1 to FBXL14, as overexpression of USP13 remarkably decreased the interaction of endogenous FBXL14 with Twist1 (Fig. 4c). Moreover, we found that the interaction between endogenous FBXL14 and Twist1 increased as a result of USP13 knockdown in SUM159PT cells (Fig. 4d). Based on the findings that FBXL14 overexpression induced Twist1 K48 ubiquitination and that co-expression of USP13 heavily prevented the appearance of Twist1 K48 ubiquitination (Fig. 4e), we conclude that USP13 can cleave K48 ubiquitin chains attached to Twist1 by FBXL14.

### USP13 promotes breast cancer cell migration, invasion and lung metastasis through Twist1

**Fig. 5:**
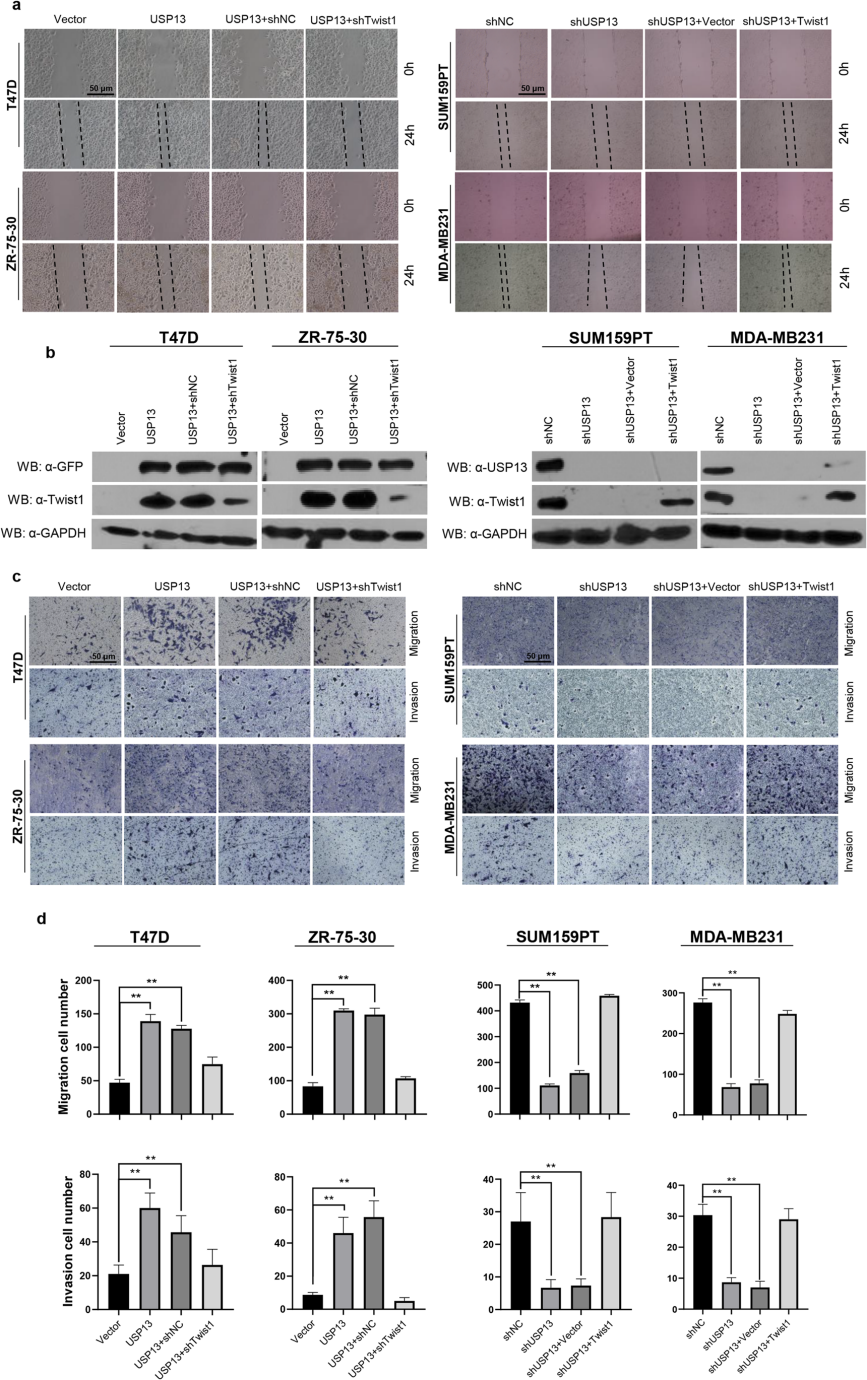
USP13 promotes breast cancer cell migration and invasion through Twist1. (**a**) Scratch wound healing assay in the indicated breast cancer cells. Scale bar = 50 μm. (**b**) Protein levels of USP13 and Twist1 in the indicated wound healing assays examined by Western blotting. (**c**) Transwell assay in the indicated breast cancer cells. Scale bar = 50 μm. (**d**) Statistical analysis of cell migration and invasion in the indicated Transwell assays**.** Data are presented as means ± SD (n = 3). Two-tailed t-test was performed. *p < 0.05; **p < 0.01

To fully explore the interplay between USP13 and Twist1, we examined the role of USP13 in inducing breast cancer cell migration and invasion through Twist1. We found that in two cell lines, T47D and ZR-75–30, which represent typical luminal cells with undetected endogenous Twist1 expression, the expression of USP13 significantly increased the levels of Twist1 and its downstream proteins (Fig. 4f). In addition, we found that overexpression of USP13 significantly changed the morphology of these two cell lines from a stationary polarity to a spindle shape, which corroborated functional activation of the EMT program (Fig. 4g). Using two specific shRNAs to knock down endogenous Twist1 expression in SUM159PT and MDA-MB231 cells (Fig. 4h), we found that USP13 expression rescued the reduced endogenous Twist1 expression caused by shTwist1 in these cells (Fig. S3a). Using scratch wound healing and Transwell assays, we found that USP13 overexpressing T47D and ZR-75–30 cells showed greater migrative and invasive capabilities than empty vector-transfected cells. Twist1 expression knockdown greatly inhibited these capabilities of USP13 overexpressing cells. On the contrary, we found that USP13 knockdown markedly decreased the migrative and invasive abilities of SUM159PT and MDA-MB231 cells, whereas the shUSP13-inhibited capabilities were restored by overexpression of Twist1 or treatment with 10 ng/ml TGF-β1, which plays a role in inducing Twist1 expression and the EMT program (Fig. 5a-d and Fig. S3b-S3e). These results underscore a key role of Twist1 in a USP13-induced enhancement of migration and invasion capacities of breast cancer cells.

**Fig. 6:**
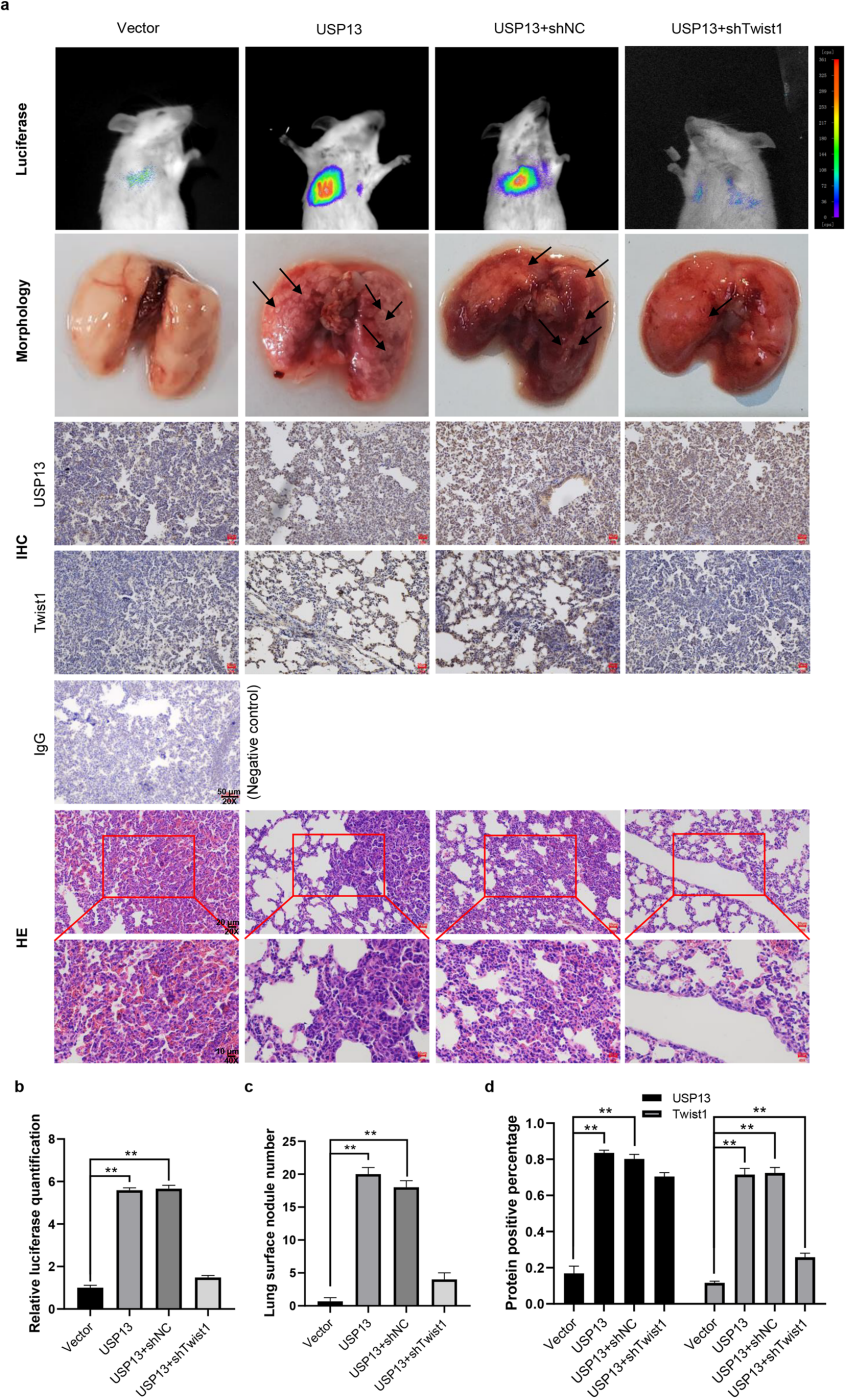
USP13 promotes breast cancer lung metastasis through Twist1. (**a**) The indicated 4T1/Luc cells were injected by the tail vein into BALB/c mice. Tumor metastasis was monitored by bioluminescence imaging. Representative images show morphology and IHC and HE staining of lung tissues from the different experimental groups. (**b**) Relative quantification of bioluminescence of the indicated mouse groups examined by photoshop software. Data are presented as means ± SD (n = 3). Two-tailed t-test was performed. *p < 0.05; **p < 0.01. (**c**) Quantification of surface nodules of the indicated mouse lungs is shown. Data are presented as means ± SD (n = 3). Two-tailed t-test was performed. *p < 0.05; **p < 0.01. (**d**) Quantification of USP13 and Twist1 positivity of the indicated mouse IHC sections examined using photoshop software. Data are presented as means ± SD (n = 3). Two-tailed t-test was performed. *p < 0.05; **p < 0.01

We also assessed the effects of USP13 expression on lung metastasis of breast cancer to further substantiate the above in vitro findings. We found that BALB/c mice injected with USP13 overexpressing 4T1/Luc cells had more lung metastatic nodules than those in the control group. The results of HE staining showed an increased proportion of nuclear atypia in lung sections of mice injected with USP13-overexpressing 4T1/Luc cells, including abnormal nuclear size and darker nuclear staining, revealing the severity of lung metastases from mammary tumors [44, 45]. Meanwhile, we detected a decrease in the number of lung metastases after injecting USP13-overexpressing 4T1/Luc cells with Twist1 expression knockdown (Fig. 6a-d and Fig. S3f-S3h), which was verified by IHC and HE analyses. These data underscore a key role of Twist1 in USP13-induced enhancement of lung metastasis in breast cancer.

### Twist1 inhibits USP13 promoter transcription

**Fig. 7:**
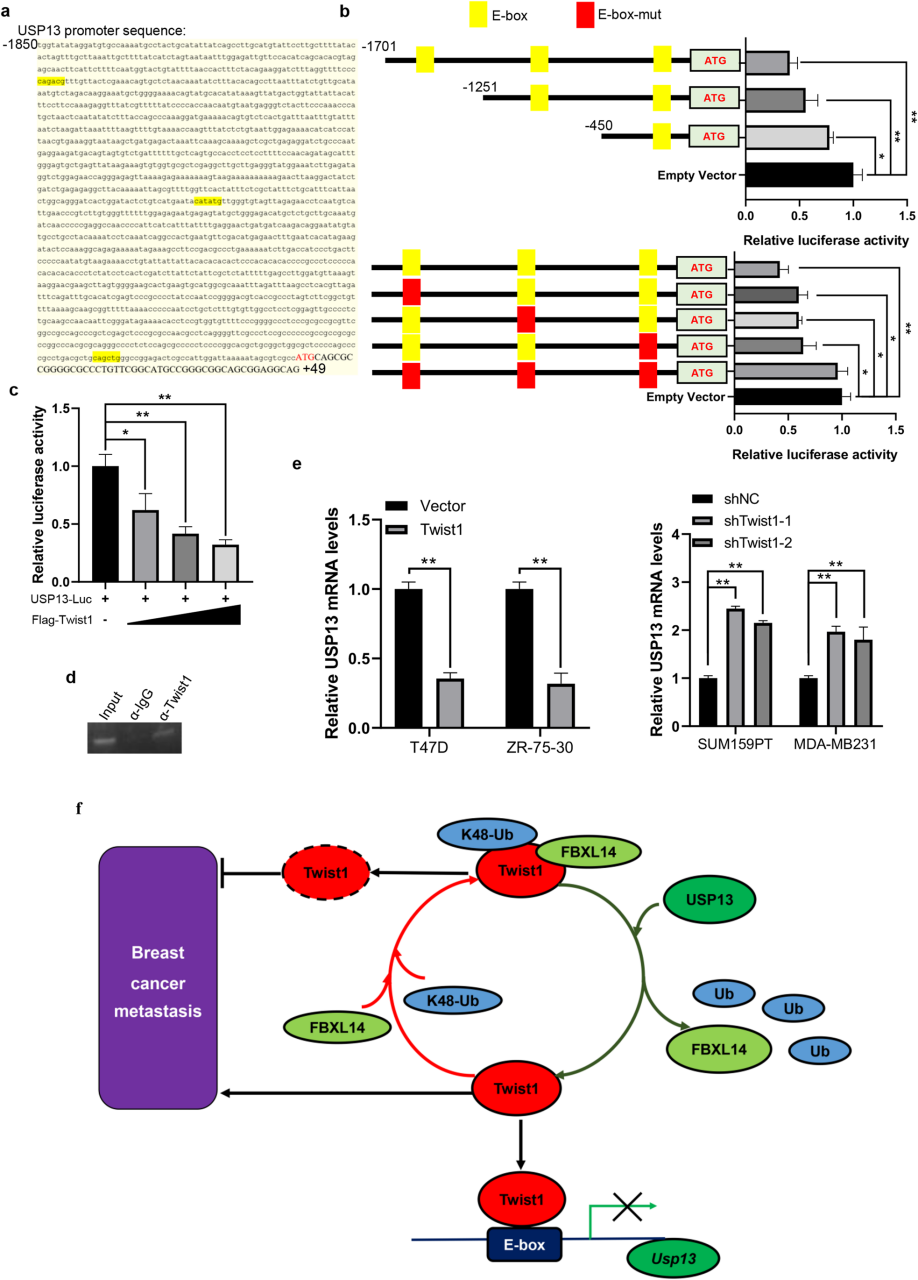
Twist1 inhibits USP13 promoter activation. (**a**) The nucleotide sequences and positions of three putative Twist1 binding site in the USP13 promoter are shown. Luciferase activity assays of the pGL3-USP13 promoter-reporter (**b**), serially truncated and mutation analysis (**c**) and dose change of Twist1 (co-transfected SUM159PT cells with USP13-Luc and 0, 0.5, 1, 2 mg Flag-Twist1). Data are presented as means ± SD (n = 3). Two-tailed t-test was performed. *p < 0.05; **p < 0.01. (**d**) ChIP assay showing direct binding of Twist1 to the USP13 promoter in SUM159PT cells. (**e**) Effects of Twist1 overexpression or knockdown on mRNA levels of USP13 in the indicated breast cancer cells. (**f**) Schematic representation of the regulatory interaction network between USP13 and Twist1 in breast cancer

With Twist1 being a transcriptional factor and sequence analysis of the USP13 promoter from position -1850 to + 49 showing three putative Twist1 binding sites (E-box, CANNTG), we assumed that Twist1 may affect the transcription of the USP13 gene (Fig. 7a). Using luciferase reporter assays in conjunction with serial deletion and site-directed mutagenesis of the USP13 promoter region, we found that three Twist1-binding sites play a pivotal role in USP13 expression (Fig. 7b). In addition, we found that Twist1 could inhibit the activity of the USP13 promoter in a dose-dependent manner (Fig. 7c), and directly bind to the USP13 promoter in SUM159PT cells as demonstrated by ChIP (Fig. 7d). Twist1 overexpression decreased USP13 mRNA levels in T47D and ZR-75–30 cells, whereas Twist1 knockdown increased USP13 mRNA levels in SUM159PT and MDA-MB231 cells (Fig. 7e). Taken together we found that, on the one hand, USP13 stabilizes Twist1 and, on the other hand, Twist1 inhibits USP13 promoter activation, thereby forming a negative feedback loop in breast cancer cells.

## Discussion

Breast cancer is the most common cancer in women worldwide, ranking second in the global female cancer mortality rate [1]. Most breast cancer-related deaths are caused primarily by the occurrence of metastasis, a process by which tumor cells disseminate through the blood or lymphatic systems to distant sites where they continue to grow [4, 46]. Abundant blood and lymphatic vessels are present in mammary glands to maintain the normal metabolism of these glands. Due to the presence and distribution characteristics of these blood and lymphatic vessels, breast cancer cells can easily metastasize after transformation. Recent studies have shown that abnormal EMT activation is closely related to the malignant transformation and metastasis of breast cancer cells [47, 48]. In addition, important pathological markers of breast cancer, such as ERα, HER2 and p53, are all related to the occurrence of EMT [49–51]. Therefore, examining the relationship between EMT and breast cancer is of great significance for revealing the mechanisms of breast cancer occurrence and progression, and may provide a theoretical basis for the treatment of breast cancer.

During the initial stages of EMT, the expression of EMT-TFs is abnormally activated, which inhibits the expression of E-cadherin and promotes tumor progression [52]. EMT-TFs play a central role in regulating important normal physiological and pathological processes, such as embryonic development, tissue fibrosis and cancer progression [53]. EMT-TFs all have noticeably short half-lives and are frequently degraded by ubiquitination in vivo. It has been reported that FBXL14 can ubiquitinate all EMT-TFs [54–56], while FBXW8 can ubiquitinate Slug and ZEB [57]. In addition to ubiquitinating the classical tumor suppressor gene p53, MDM2 can also ubiquitinate Slug [58]. Also, β-TRCP1 can ubiquitinate Snail and Twist, but the latter two need to be phosphorylated by GSK3β [57] and IKKβ [17], respectively. In addition, several discoveries have been made about the de-ubiquitination of EMT-TFs, i.e., Dub3 de-ubiquitinates Snail [59], Slug and Twist [22], whereas Trabid stabilizes Twist by cleaving RNF8-induced ubiquitination [21] and USP29 cooperates with phosphatase SCP1 to stabilize Snail [60].

As an important EMT-TF, Twist1 is highly expressed in breast cancer patients. Its expression is further elevated in highly aggressive triple-negative breast cancer cells compared to other breast cancer cells [61]. Next to Twist1, the transcription factor c-Myc is a member of the BHLH family that plays an essential role in cell proliferation. It has been reported that c-Myc can be de-ubiquitinated by USP13 [33]. In addition, USP13 has been reported to de-ubiquitinate another EMT-TF, Snail [62]. Therefore, we set out to investigate whether USP13 may affect Twist1. USP13 is orthologous to USP5 and shows ~ 80% sequence similarity with USP5 [63]. The USP13 gene is located on a different chromosome than USP5 and also exhibits a different expression profile, with USP13 showing higher levels of de-ubiquitination and Ub activation activities. The tandem UBA domains of USP13 are more likely to bind to polyUb chains to improve hydrolysis efficiency. It has been shown that USP13-ZnF does not bind Ub, while USP5-ZnF binds Ub with a relatively high affinity. Therefore, differential recognition of Ub by the ZnF domain may provide a molecular basis for the differentially regulated catalysis of these two enzymes [64]. Our study supports this finding by validating that USP5 does not have the same effect on Twist1 as USP13. Furthermore, USP13 may be associated with other ubiquitin-like (UbL) proteins such as ISG15, SUMO1 and SUMO2 [65, 66]. Based on these findings, it is assumed that the de-ubiquitinating activity of USP13 and the Ub-binding capacity of its UBA domain may play essential roles in regulating cellular protein levels. We found that USP13 de-ubiquitinates Twist1 by binding to the WR region. USP13 could cleave FBXL14-induced Twist1 K48 ubiquitination to stabilize Twist1, but it was ineffective in Twist1 K63 ubiquitination. This result contradicts Zhang et al.’s claim that USP13 can hydrolyze both K48 and K63-linked Ub chains with high efficiency, but preferentially hydrolyzes K63-linked poly Ub chains [67]. Knockdown of Twist1 reversed the migration and invasion capacities of breast cancer cells and the occurrence of lung metastasis in mice caused by overexpression of USP13. Moreover, USP13 had no effect on the ubiquitination of Twist1 by β-Trcp1, presumably due to the ineffectiveness of USP13 in Twist1 phosphorylation.

As a typical transcription factor, Twist1 can recognize and bind to the E-box region of downstream gene promoters to activate or inhibit the transcription of target genes [68]. By analyzing the USP13 promoter sequence, we identified three E-box regions. Drawn from the combined results from the ChIP and luciferase reporter assays, we found that Twist1 can inhibit the transcription of USP13 by directly binding to these three E-box regions. Interestingly, Twist1 tends to recruit other co-factors to affect target gene transcription synergistically, such as RNA polymerase II and histone acetyltransferase. Actually, we found that the expression of USP13 promotes the interaction between Twist1 and BRD4 (data not shown), which can induce Twist1 to recruit RNA polymerase II to promote downstream gene transcription [14]. However, this finding is contradictory to the notion that Twist1 inhibits USP13 promoter activation. A possible, but one-sided explanation may be that normal physiological concentrations of Twist1 individually inhibit USP13 promoter activation, whereas abnormal expression of USP13 may induce Twist1 to recruit co-factors to promote the transcription of its target genes including USP13. The putative molecular mechanisms underlying this phenomenon require further experimental verification.

## Conclusions

We found that USP13 and Twist1 can form a negative feedback loop to jointly regulate the migration and invasion of breast cancer cells. This finding may facilitate the identification of new therapeutic targets to treat patients with breast cancer.

## Supplementary Information

Below is the link to the electronic supplementary material.

Supplementary file1 (PDF 792 KB)

## Data Availability

The authors declare that the data supporting the findings of this study are available within the paper. The data analysis of the expression levels of USP13 in normal breast tissues and different subclasses of carcinomatous breast tissues are available in ULACAN (http://ualcan.path.uab.edu/), and the Kaplan–Meier curves that support the findings of this study are available in the UCSC Xena database (https://xenabrowser.net/) and analyzed by the R package survival and survminer tools.

## References

[1] Siegel RL, Miller KD, Fuchs HE, Jemal A (2022). Cancer statistics, 2022. CA Cancer J. Clin..

[2] Festari MF, da Costa V, Rodríguez-Zraquia SA, Costa M, Landeira M, Lores P, Solari-Saquieres P, Kramer MG, Freire T (2022). The tumor-associated Tn antigen fosters lung metastasis and recruitment of regulatory T cells in triple negative breast cancer. Glycobiology.

[3] Lambert AW, Pattabiraman DR, Weinberg RA (2017). Emerging biological principles of metastasis. Cell.

[4] Roche J (2018). Erratum: Roche J. The epithelial-to-mesenchymal transition in cancer. Cancers.

[5] Barnes RM, Firulli AB (2009). A twist of insight - the role of Twist-family bHLH factors in development. Int. J. Dev. Biol..

[6] Zhao Z, Rahman MA, Chen ZG, Shin DM (2017). Multiple biological functions of Twist1 in various cancers. Oncotarget.

[7] Kalluri R, Neilson EG (2003). Epithelial-mesenchymal transition and its implications for fibrosis. J. Clin. Invest..

[8] Zhu Q-Q, Ma C, Wang Q, Song Y, Lv T (2016). The role of TWIST1 in epithelial-mesenchymal transition and cancers. Tumour Biol..

[9] Firulli BA, Krawchuk D, Centonze VE, Vargesson N, Virshup DM, Conway SJ, Cserjesi P, Laufer E, Firulli AB (2005). Altered Twist1 and Hand2 dimerization is associated with Saethre-Chotzen syndrome and limb abnormalities. Nat. Genet..

[10] Vichalkovski A, Gresko E, Hess D, Restuccia DF, Hemmings BA (2010). PKB/AKT phosphorylation of the transcription factor Twist-1 at Ser42 inhibits p53 activity in response to DNA damage. Oncogene.

[11] Li C-W, Xia W, Lim S-O, Hsu JL, Huo L, Wu Y, Li L-Y, Lai C-C, Chang S-S, Hsu Y-H, Sun H-L, Kim J, Yamaguchi H, Lee D-F, Wang H, Wang Y, Chou C-K, Hsu J-M, Lai Y-J, LaBaff AM, Ding Q, Ko H-W, Tsai F-J, Tsai C-H, Hortobagyi GN, Hung M-C (2016). AKT1 inhibits epithelial-to-mesenchymal transition in breast cancer through phosphorylation-dependent Twist1 degradation. Cancer Res..

[12] Hong J, Zhou J, Fu J, He T, Qin J, Wang L, Liao L, Xu J (2011). Phosphorylation of serine 68 of Twist1 by MAPKs stabilizes Twist1 protein and promotes breast cancer cell invasiveness. Cancer Res..

[13] Su Y-W, Xie T-X, Sano D, Myers JN (2011). IL-6 stabilizes Twist and enhances tumor cell motility in head and neck cancer cells through activation of casein kinase 2. PLoS ONE.

[14] Shi J, Wang Y, Zeng L, Wu Y, Deng J, Zhang Q, Lin Y, Li J, Kang T, Tao M, Rusinova E, Zhang G, Wang C, Zhu H, Yao J, Zeng Y-X, Evers BM, Zhou M-M, Zhou BP (2014). Disrupting the interaction of BRD4 with diacetylated Twist suppresses tumorigenesis in basal-like breast cancer. Cancer Cell.

[15] Gajula RP, Chettiar ST, Williams RD, Thiyagarajan S, Kato Y, Aziz K, Wang R, Gandhi N, Wild AT, Vesuna F, Ma J, Salih T, Cades J, Fertig E, Biswal S, Burns TF, Chung CH, Rudin CM, Herman JM, Hales RK, Raman V, An SS, Tran PT (2013). The twist box domain is required for Twist1-induced prostate cancer metastasis. Mol. Cancer Res..

[16] Zhong J, Ogura K, Wang Z, Inuzuka H (2013). Degradation of the transcription factor Twist, an oncoprotein that promotes cancer metastasis. Discov. Med..

[17] Lander R, Nordin K, LaBonne C (2011). The F-box protein Ppa is a common regulator of core EMT factors Twist, Snail, Slug, and Sip1. J. Cell. Biol..

[18] Lee H-J, Li C-F, Ruan D, Powers S, Thompson PA, Frohman MA, Chan C-H (2016). The DNA damage transducer RNF8 facilitates cancer chemoresistance and progression through Twist activation. Mol. Cell.

[19] Chen H, Hu L, Luo Z, Zhang J, Zhang C, Qiu B, Dong L, Tan Y, Ding J, Tang S, Shen F, Li Z, Wang H (2015). A20 suppresses hepatocellular carcinoma proliferation and metastasis through inhibition of Twist1 expression. Mol. Cancer.

[20] Zhu Y, Qu C, Hong X, Jia Y, Lin M, Luo Y, Lin F, Xie X, Xie X, Huang J, Wu Q, Qiu X, Piao D, Xing Y, Yu T, Lu Y, Huang Q, Yu C, Jin J, Zhang Z (2019). Trabid inhibits hepatocellular carcinoma growth and metastasis by cleaving RNF8-induced K63 ubiquitination of Twist1. Cell Death Differ..

[21] Lin Y, Wang Y, Shi Q, Yu Q, Liu C, Feng J, Deng J, Evers BM, Zhou BP, Wu Y (2017). Stabilization of the transcription factors slug and twist by the deubiquitinase dub3 is a key requirement for tumor metastasis. Oncotarget.

[22] Qiang L, Zhao B, Ming M, Wang N, He T-C, Hwang S, Thorburn A, He Y-Y (2014). Regulation of cell proliferation and migration by p62 through stabilization of Twist1. PNAS.

[23] Reyes-Turcu FE, Horton JR, Mullally JE, Heroux A, Cheng X, Wilkinson KD (2006). The ubiquitin binding domain ZnF UBP recognizes the C-terminal diglycine motif of unanchored ubiquitin. Cell.

[24] Liu J, Xia H, Kim M, Xu L, Li Y, Zhang L, Cai Y, Norberg HV, Zhang T, Furuya T, Jin M, Zhu Z, Wang H, Yu J, Li Y, Hao Y, Choi A, Ke H, Ma D, Yuan J (2011). Beclin1 controls the levels of p53 by regulating the deubiquitination activity of USP10 and USP13. Cell.

[25] Zhang J, Zhang P, Wei Y, Piao H-L, Wang W, Maddika S, Wang M, Chen D, Sun Y, Hung M-C, Chen J, Ma L (2013). Deubiquitylation and stabilization of PTEN by USP13. Nat. Cell. Biol..

[26] Man X, Piao C, Lin X, Kong C, Cui X, Jiang Y (2019). USP13 functions as a tumor suppressor by blocking the NF-kB-mediated PTEN downregulation in human bladder cancer. J. Exp. Clin. Cancer Res..

[27] Qu Z, Zhang R, Su M, Liu W (2019). USP13 serves as a tumor suppressor via the PTEN/AKT pathway in oral squamous cell carcinoma. Cancer Manag. Res..

[28] Xiang S, Fang J, Wang S, Deng B, Zhu L (2015). MicroRNA-135b regulates the stability of PTEN and promotes glycolysis by targeting USP13 in human colorectal cancers. Oncol. Rep..

[29] Kwon J, Choi H, Ware AD, Morillo BC, Wang H, Bouker KB, Lu X, Waldman T, Han C (2022). USP13 promotes development and metastasis of high-grade serous ovarian carcinoma in a novel mouse model. Oncogene.

[30] Huang J, Ye Z, Wang J, Chen Q, Huang D, Liu H (2021). USP13 mediates PTEN to ameliorate osteoarthritis by restraining oxidative stress, apoptosis and inflammation via AKT-dependent manner. Biomed. Pharmacother..

[31] Geng J, Huang X, Li Y, Xu X, Li S, Jiang D, Liang J, Jiang D, Wang C, Dai H (2015). Down-regulation of USP13 mediates phenotype transformation of fibroblasts in idiopathic pulmonary fibrosis. Respir. Res..

[32] Zhao X, Fiske B, Kawakami A, Li J, Fisher DE (2011). Regulation of MITF stability by the USP13 deubiquitinase. Nat. Commun..

[33] Fang X, Zhou W, Wu Q, Huang Z, Shi Y, Yang K, Chen C, Xie Q, Mack SC, Wang X, Carcaboso AM, Sloan AE, Ouyang G, McLendon RE, Bian X-W, Rich JN, Bao S (2017). Deubiquitinase USP13 maintains glioblastoma stem cells by antagonizing FBXL14-mediated Myc ubiquitination. J. Exp. Med..

[34] Sun H, Zhang Q, Jing Y-Y, Zhang M, Wang H-Y, Cai Z, Liuyu T, Zhang Z-D, Xiong T-C, Wu Y, Zhu Q-Y, Yao J, Shu H-B, Lin D, Zhong B (2017). USP13 negatively regulates antiviral responses by deubiquitinating STING. Nat. Commun..

[35] Li Y, Luo K, Yin Y, Wu C, Deng M, Li L, Chen Y, Nowsheen S, Lou Z, Yuan J (2017). USP13 regulates the RAP80-BRCA1 complex dependent DNA damage response. Nat. Commun..

[36] Jia Z, Wang M, Li S, Li X, Bai X-Y, Xu Z, Yang Y, Li B, Li Y, Wu H (2018). U-box ubiquitin ligase PPIL2 suppresses breast cancer invasion and metastasis by altering cell morphology and promoting SNAI1 ubiquitination and degradation. Cell Death Dis..

[37] Xu Z, Yang Y, Li B, Li Y, Xia K, Yang Y, Li X, Wang M, Li S, Wu H (2018). Checkpoint suppressor 1 suppresses transcriptional activity of ERα and breast cancer cell proliferation via deacetylase SIRT1. Cell Death Dis..

[38] Zhao F, Wang M, Li S, Bai X, Bi H, Liu Y, Ao X, Jia Z, Wu H (2015). DACH1 inhibits SNAI1-mediated epithelial-mesenchymal transition and represses breast carcinoma metastasis. Oncogenesis.

[39] Bi H, Li S, Qu X, Wang M, Bai X, Xu Z, Ao X, Jia Z, Jiang X, Yang Y, Wu H (2015). DEC1 regulates breast cancer cell proliferation by stabilizing cyclin E protein and delays the progression of cell cycle S phase. Cell Death Dis..

[40] Schmittgen TD, Livak KJ (2008). Analyzing real-time PCR data by the comparative C(T) method. Nat. Protoc..

[41] Chandrashekar DS, Bashel B, Balasubramanya SAH, Creighton CJ, Ponce-Rodriguez I, Chakravarthi BVSK, Varambally S (2017). UALCAN: A portal for facilitating tumor subgroup gene expression and survival analyses. Neoplasia.

[42] Terry MT, Patricia MG (2000). Modeling survival data: Extending the Cox model.

[43] K. Alboukadel, K. Marcin, B. Przemyslaw, Survminer: Drawing survival curves using 'ggplot2'. R package version 0.4.9. (2021)

[44] Rui Y, Ren F, Li JT, Rao XS, Lv ZL, Zheng CH, Zhang F (2022). Nuclei-guided network for breast cancer grading in he-stained pathological images. Sensors (Basel)..

[45] Khan AM, Sirinukunwattana K, Rajpoot N (2015). A global covariance descriptor for nuclear atypia scoring in breast histopathology images. IEEE J. Biomed. Health Inform..

[46] Yu M, Bardia A, Wittner BS, Stott SL, Smas ME, Ting DT, Isakoff SJ, Ciciliano JC, Wells MN, Shah AM, Concannon KF, Donaldson MC, Sequist LV, Brachtel E, Sgroi D, Baselga J, Ramaswamy S, Toner M, Haber DA, Maheswaran S (2013). Circulating breast tumor cells exhibit dynamic changes in epithelial and mesenchymal composition. Science.

[47] Zhao Y, Sun H, Zhao Y, Liu Q, Liu Y, Hou Y, Jin W (2022). NSrp70 suppresses metastasis in triple-negative breast cancer by modulating Numb/TβR1/EMT axis. Oncogene.

[48] X. Yin, X. Teng, T. Ma, T. Yang, J. Zhang, M. Huo, W. Liu, Y. Yang, B. Yuan, H. Yu, W. Huang, Y. Wang, RUNX2 recruits the NuRD(MTA1)/CRL4B complex to promote breast cancer progression and bone metastasis [published online ahead of print, 2022 May 9]. Cell Death Differ. (2022) 10.1038/s41418-022-01010-2 PMC9613664 35534547

[49] Xia X, Huang C, Liao Y, Liu Y, He J, Shao Z, Hu T, Yu C, Jiang L, Liu J, Huang H (2021). The deubiquitinating enzyme USP15 stabilizes ERα and promotes breast cancer progression. Cell Death Dis..

[50] Carpenter RL, Paw I, Dewhirst MW, Lo H-W (2015). Akt phosphorylates and activates HSF-1 independent of heat shock, leading to Slug overexpression and epithelial-mesenchymal transition (EMT) of HER2-overexpressing breast cancer cells. Oncogene.

[51] L. J. van Weele, S. I. Djomehri, S. Cai, J. Antony, S. S. Sikandar, D. Qian, W. H. D. Ho, R. B. West, F. A. Scheeren, M. F. Clarke, Mesenchymal tumor cells drive adaptive resistance of Trp53-/- breast tumor cells to inactivated mutant Kras [published online ahead of print, 2022 Apr 9]. Mol Oncol. (2022) 10.1002/1878-0261.13220 PMC9441006 35398967

[52] Jordan NV, Johnson GL, Abell AN (2011). Tracking the intermediate stages of epithelial-mesenchymal transition in epithelial stem cells and cancer. Cell Cycle.

[53] Huang RY-J, Guilford P, Thiery JP (2012). Early events in cell adhesion and polarity during epithelial-mesenchymal transition. J. Cell Sci..

[54] Voutsadakis IA (2012). Epithelial to mesenchymal transition in the pathogenesis of uterine malignant mixed Müllerian tumours: the role of ubiquitin proteasome system and therapeutic opportunities. Clin. Transl. Oncol..

[55] Voutsadakis IA (2012). The ubiquitin-proteasome system and signal transduction pathways regulating Epithelial Mesenchymal transition of cancer. J. Biomed. Sci..

[56] Voutsadakis IA (2012). Ubiquitination and the Ubiquitin-Proteasome System as regulators of transcription and transcription factors in epithelial mesenchymal transition of cancer. Tumour Biol..

[57] Fu J, Lv X, Lin H, Wu L, Wang R, Zhou Z, Zhang B, Wang Y-L, Tsang BK, Zhu C, Wang H (2010). Ubiquitin ligase cullin 7 induces epithelial-mesenchymal transition in human choriocarcinoma cells. J. Biol. Chem..

[58] Wang S-P, Wang W-L, Chang Y-L, Wu C-T, Chao Y-C, Kao S-H, Yuan A, Lin C-W, Yang S-C, Chan W-K, Li K-C, Hong T-M, Yang P-C (2009). p53 controls cancer cell invasion by inducing the MDM2-mediated degradation of Slug. Nat. Cell. Biol..

[59] Zhou BP, Deng J, Xia W, Xu J, Li YM, Gunduz M, Hung M-C (2004). Dual regulation of Snail by GSK-3beta-mediated phosphorylation in control of epithelial-mesenchymal transition. Nat. Cell. Biol..

[60] Wu Y, Wang Y, Lin Y, Liu Y, Wang Y, Jia J, Singh P, Chi Y-I, Wang C, Dong C, Li W, Tao M, Napier D, Shi Q, Deng J, Evers BM, Zhou BP (2017). Dub3 inhibition suppresses breast cancer invasion and metastasis by promoting Snail1 degradation. Nat. Commun..

[61] Kallergi G, Papadaki MA, Politaki E, Mavroudis D, Georgoulias V, Agelaki S (2011). Epithelial to mesenchymal transition markers expressed in circulating tumour cells of early and metastatic breast cancer patients. Breast Cancer Res..

[62] Zhang T, Zheng J, Qiao L, Zhao W (2022). Deubiquitinase USP13 promotes the epithelial-mesenchymal transition and metastasis in gastric cancer by maintaining Snail protein. Pathol. Res. Pract..

[63] Qian W, Li Q, Wu X, Li W, Li Q, Zhang J, Li M, Zhang D, Zhao H, Zou X, Jia H, Zhang L, Yang X-D, Hou Z (2020). Deubiquitinase USP29 promotes gastric cancer cell migration by cooperating with phosphatase SCP1 to stabilize Snail protein. Oncogene.

[64] Xie X, Matsumoto S, Endo A, Fukushima T, Kawahara H, Saeki Y, Komada M (2018). Deubiquitylases USP5 and USP13 are recruited to and regulate heat-induced stress granules through their deubiquitylating activities. J. Cell Sci..

[65] Catic A, Fiebiger E, Korbel GA, Blom D, Galardy PJ, Ploegh HL (2007). Screen for ISG15-crossreactive deubiquitinases. PLoS ONE.

[66] Ghosh DK, Ranjan A (2021). HYPK coordinates degradation of polyneddylated proteins by autophagy. Autophagy.

[67] Zhang Y-H, Zhou C-J, Zhou Z-R, Song A-X, Hu H-Y (2011). Domain analysis reveals that a deubiquitinating enzyme USP13 performs non-activating catalysis for Lys63-linked polyubiquitin. PLoS ONE.

[68] Peinado H, Olmeda D, Cano A (2007). Snail, Zeb and bHLH factors in tumour progression: an alliance against the epithelial phenotype?. Nat. Rev. Cancer..

