# Supplementary material for: USP13 promotes breast cancer metastasis through FBXL14-induced Twist1 ubiquitination
Source: Cell Oncol (Dordr). 2023 Feb 3;46(3):717–33. doi: 10.1007/s13402-023-00779-9 (PMC10205858; doi:10.1007/s13402-023-00779-9)
Supplement: Supplementary file 1 — Supplementary file1 (PDF 792 KB) [file 13402_2023_779_MOESM1_ESM.pdf]

Supplementary Information

Table S1 Statistical analysis of the expression level of USP13 in normal breast tissues and three subclasses breast carcinomatous tissues in the TCGA database

Comparison	Statistical significance
Normal-vs-Luminal	1.10E-01
Normal-vs-HER2 Positive	9.26E-02
Normal-vs-TNBC	4.29E-01
Luminal-vs-HER2 Positive	4.95E-01
Luminal-vs-TNBC	1.70E-05 **
HER2 Positive-vs-TNBC	4.02E-03 **

Fig.S1 USP13 stabilizes Twist1 protein. The Kaplan–Meier curves obtained from UCSC Xena database and analyzed by the R package survival and survminer.

Fig.S2 USP13 deubiquitinates Twist1. (**a**) Flag-Twist1 was co-expressed with GFP-USP13 or empty vector in HEK293T cells and treated with 20 μM MG132. The relative mRNA levels of Twist1 and USP13 were determined using qRT-PCR. (**b**) GFP-USP13 was expressed with increasing amounts in MDA-MB231 cells. The relative mRNA levels of Twist1 and USP13 were determined using qRT-PCR. (**c**) SUM159PT and MDA-MB231 cells were stably transfected with shNC control or USP13 shRNAs and the relative mRNA levels of Twist1, c-Myc and USP13 were determined using qRT-PCR. (**d-g**) Grayscale analysis of Fig.3a-3d revealed Twist1 relative protein levels in indicated CHX experiments. Data are means ± SD (n = 3). Two-tailed t-test was performed. *$p < 0.05$; **$p < 0.01$. (**h,i**) Grayscale analysis of Fig.4a revealed Twist1 K48 or K63 ubiquitination changes in the presence or absence of GFP-USP13. Data are means ± SD (n = 3). Two-tailed t-test was performed. *$p < 0.05$; **$p < 0.01$.

Fig.S3 USP13 promotes breast cancer cell migration, invasion and lung metastasis through Twist1. (**a**) SUM159PT and MDA-MB231 cells were transfected with indicated plasmids and then western blot was performed. (**b**) The wound healing assay in indicated breast cancer cells treated with or without 10ng/mL TGF-β1. Scale bar = 50 μm. (**c**) The protein levels of USP13 and Twist1 in indicated wound healing assays were examined by western blot. (**d**) The Transwell assay in indicated breast cancer cells treated with or without 10ng/mL TGF-β1. Scale bar = 50 μm. (**e**) Statistical analysis of migration and invasion cells in indicated Transwell assays**.** Data are means ± SD (n = 3). Two-tailed t-test was performed. *$p < 0.05$; **$p < 0.01$. (**f**) Representative images showing morphology of lung tissues from the different experimental groups. (**g**) The protein levels of USP13 and Twist1 in indicated mouse groups were examined by western blot. (**h**) Lung weight of mice in different groups. Data are means ± SD (n = 3). Two-tailed t-test was performed. *$p < 0.05$; **$p < 0.01$.

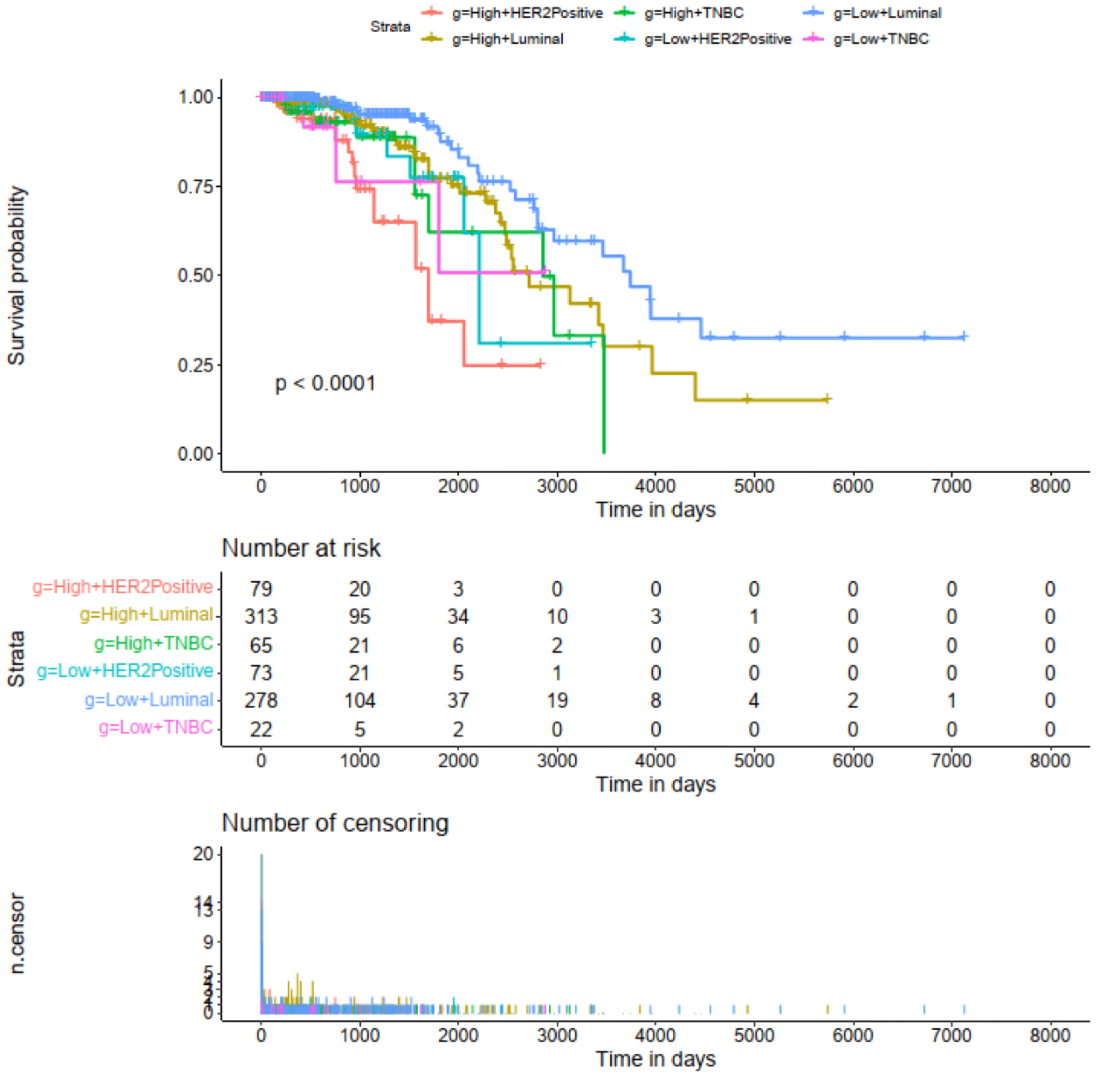
Strata
g=High+HER2Positive
g=High+Luminal
g=High+TNBC
g=Low+HER2Positive
g=Low+Luminal
g=Low+TNBC
Survival probability
1.00
0.75
0.50
0.25
0.00
p < 0.0001
0 1000 2000 3000 4000 5000 6000 7000 8000
Time in days
Number at risk
Strata
g=High+HER2Positive 79 20 3 0 0 0 0 0 0
g=High+Luminal 313 95 34 10 3 1 0 0 0
g=High+TNBC 65 21 6 2 0 0 0 0 0
g=Low+HER2Positive 73 21 5 1 0 0 0 0 0
g=Low+Luminal 278 104 37 19 8 4 2 1 0
g=Low+TNBC 22 5 2 0 0 0 0 0 0
0 1000 2000 3000 4000 5000 6000 7000 8000
Time in days
Number of censoring
n.censor
20
9
0
0 1000 2000 3000 4000 5000 6000 7000 8000
Time in days

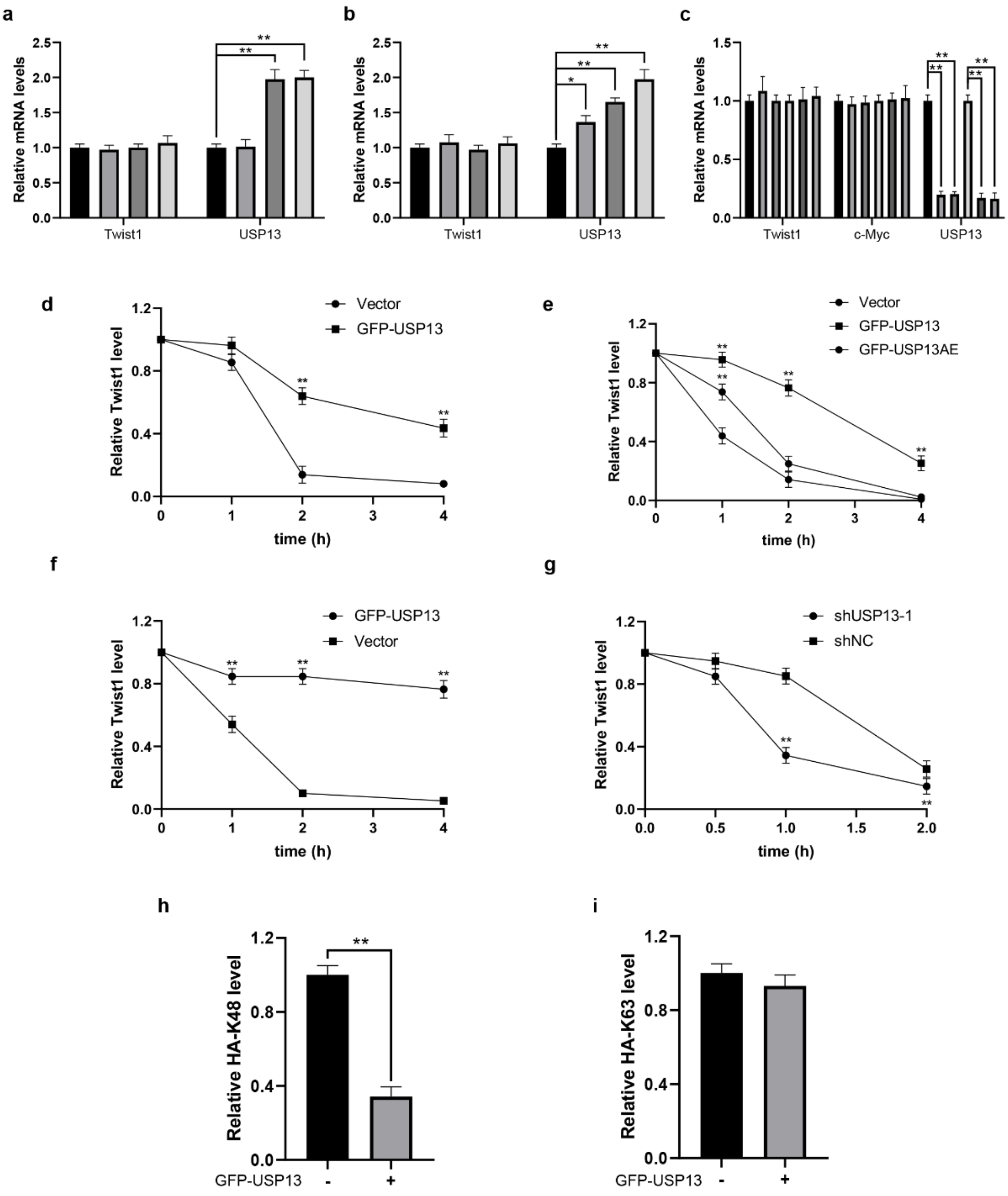
a
Relative mRNA levels
Twist1
USP13
b
Relative mRNA levels
Twist1
USP13
c
Relative mRNA levels
Twist1
c-Myc
USP13
d
Vector
GFP-USP13
Relative Twist1 level
time (h)
e
Vector
GFP-USP13
GFP-USP13AE
Relative Twist1 level
time (h)
f
GFP-USP13
Vector
Relative Twist1 level
time (h)
g
shUSP13-1
shNC
Relative Twist1 level
time (h)
h
Relative HA-K48 level
GFP-USP13
i
Relative HA-K63 level
GFP-USP13

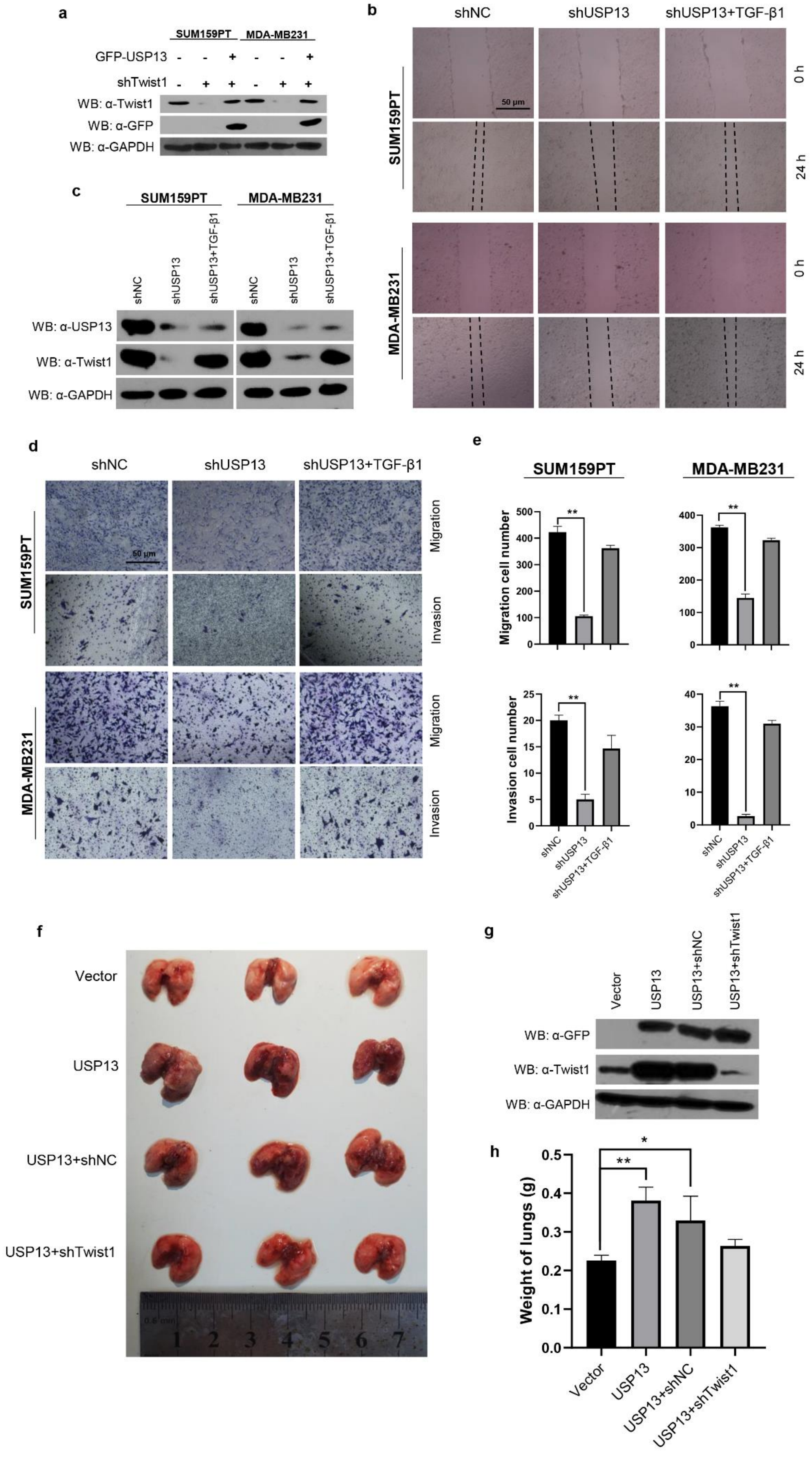
a
SUM159PT
MDA-MB231
GFP-USP13
shTwist1
WB: α-Twist1
WB: α-GFP
WB: α-GAPDH
b
shNC
shUSP13
shUSP13+TGF-β1
SUM159PT
MDA-MB231
0 h
24 h
50 μm
c
shNC
shUSP13
shUSP13+TGF-β1
WB: α-USP13
WB: α-Twist1
WB: α-GAPDH
d
Migration
Invasion
e
Migration cell number
Invasion cell number
f
Vector
USP13
USP13+shNC
USP13+shTwist1
g
WB: α-GFP
h
Weight of lungs (g)
